# Is there a common pattern of dental specialties in the world? Orthodontics, the constant element

**DOI:** 10.1186/s12903-023-03713-5

**Published:** 2024-01-08

**Authors:** Ignacio Garcia-Espona, Cristina Garcia-Espona, José Antonio Alarcón, Eugenia Garcia-Espona, Javier Fernández-Serrano

**Affiliations:** 1 Department of Stomatology, Section of Orthodontics, School of Dentistry, Campus Universitario de Cartuja S/N, University of Granada, 18071 Granada, Spain; 2 President of the Spanish Association of Orthodontists (AESOR), Madrid, Spain

**Keywords:** Dental specialties, Orthodontics, Oral surgery, Cluster analysis

## Abstract

**Background:**

There is a lack of studies comparing the status of dental specialties worldwide. Therefore, this study aimed to analyze the differences and similarities between the number and types of dental specialties in 31 countries, including every continent, in the world.

**Materials and methods:**

Available official documents and webpages from regulatory bodies, official colleges and councils, and dental institutions were collected from 31 countries and analyzed to obtain reliable data on dental specialties. Differences were analyzed using the Lorentz curve and Gini test. Additionally, a cluster analysis was performed to obtain groups of countries with similar patterns in the number and types of dental specialties.

**Results:**

A total of 32 different specialties were officially recognized among all the analyzed countries. Orthodontics and oral surgery (100% and 93.1%, respectively) were the two most frequently officially recognized dental specialties worldwide. The total global degree of inequality in the 31 analyzed countries was 42.4%. The Anglo-Saxon countries showed the greatest similarity, approximately 15-fold higher than the European countries. Cluster analysis differentiated six main groups of countries according to the number and types of dental specialties. European countries formed one of the two largest clusters, and the other cluster was of Anglo-Saxon, Asian, African, and several Eastern European countries with a high number of specialties.

**Conclusions:**

Officially recognized dental specialties in the different continents and countries show an asymmetric organization. The number, names, and skills of officially recognized dental specialties exhibited significant differences, showing inequalities in their organization. The Anglo-Saxon pattern of dental specialties showed greater equality than the European pattern. Orthodontics was the only constant element among the different patterns.

## Background

Orthodontics, the first dental specialty in the world, was recognized in the United States in 1900 and set a precedent in dentistry [1]. Since then, specialty training in orthodontics has been an important dental discipline, and the regulation of its postgraduate training is clearly defined in Europe based on the Erasmus Project [2].

Oral surgery is also a traditional and significant area of specialization in dentistry [3]. Orthodontics and oral surgery were the first two officially recognized dental specialties in Europe in three directives released in 1978, and their regulations were completed through the 2005/36/CE [4] and 2013/55/UE directives [5].

Subsequently, several dental specialties have been established. Every analyzed country has implemented different steps, approving and regularizing new specialties when needed. These events caused asymmetry in terms of officially recognized dental specialties [6], influenced by different factors, such as political, cultural, and social development of each country; different health systems; types of law organization systems; and requirements in dentistry.

There is a need for specialized dental care for various reasons, especially to improve the quality of treatment and patient safety [7]. Postgraduate education is also associated with higher research activity [8]. However, it is necessary to expand dental specialist training to conform with common standards for specialist education [9].

In a previous study analyzing the differences between 21 European countries, the status of dental specialties in the European Economic Area and the United Kingdom showed an unequal organization; the number and names of officially recognized dental specialties across member states were quite different, despite having some common directives [10].

Documents or data from national associations of dentists can provide relevant information to better understand the status of dental specialties in different countries. However, a study comparing the actual panorama between countries worldwide has not yet been reported. Therefore, the present study aimed to examine the worldwide differences in dental specialties from clinical and academic perspectives.

## Materials and methods

This was a descriptive, observational, and retrospective study based on information obtained from different webpages and official public documents from 31 countries, most of which were linked to dental institutions, regulatory bodies, colleges, and councils (Table 1) [11–21].

**Table 1:** Source of information, name of document or website address and year for every country^a^

Country	Source of information	WEBPAGES	Documents	
			**Name**	**Year**
Australia	Australian Dental Council (ADC)	https://adc.org.au	Annual Report 2020/21	2021
Brasil	Conselho Federal de Odontologia (CFO)	https://website.cfo.org.br	Database	2023
Canada	The National Dental Examining Board of Canada (NDEB)	https://ndeb-bned.ca	National Dental Specialty Examination (NDSE) Protocol	2022
Indonesia	Persatuan Dokter Gigi Indonesia (PDGI)	https://pdgi.or.id	Database	2023
Japan	Japan Dental Association (JDA)	https://www.jda.or.jp/	About the current situation of major specialists	2015
New Zealand	Dental Council of New Zealand	https://www.dcnz.org.nz	Annual Report 2020/21	2021
Russia	Dental Association of Russia	https://e-stomatology.ru	On the approval of the list specialties and areas of higher education training	2022
South Africa	The South African Dental Association (SADA)	https://www.sada.co.za/home	Patient education information brought to you by SADA. Meet the dentist & dental specialist	2021
Turkey	Türk Dişhekimleri Birliği (TDB) (Turkish Dental Association)	https://www.tdb.org.tr/index.php	Bazi Kanun ve Kanun Hükmünde Kararnamelerde Değişiklik yapilmasina dair Kanun (In some Law and declarations.Law on change)	2011
USA	American Dental Association (ADA)	https://www.ada.org	National Commission on Recognition of Dental Specialties and Certifying Boards	2022

^a^Data for the remaining countries analysed are cited in Garcia-Espona et al., 2023 [10]

Countries with international relevance, free access, and fully reliable information related to officially recognized dental specialties from every continent were included: European, belonging to the European Economic Area (21) (data showed in a previous study [10]), American (3), Asian (4), African (1), and Oceanian (2).

The obtained data were analyzed to compare the number, types, and patterns of dental specialties. To quantify the degree of global inequality related to the number of dental specialties in the European, non-European, and Anglo-Saxon countries, as well as the whole world, the Lorentz curve and Gini index were applied, as described in our previous study [10].

The Lorentz curve was obtained when the relationship between the cumulative percentage of countries in the world (or every group) and the cumulative percentage of specialties was graphically displayed. This curve aids in assessing inequality between countries, the closer it is to the diagonal, the more homogeneous the number of specialties in the countries; however, if it approaches the horizontal axis, more inequality exists in the number of specialties in different countries.

This property can be quantified by simply measuring the area between both curves and scale it such that the result remains between 0 and 1; as a result, the Gini (G) index is obtained. A G value close to 0 indicates high equality in the distribution, whereas a G value close to 1 indicates high inequality [22].

To assess inequality between countries and establish groups of inequality and similarity, we performed a cluster analysis, which specializes in exploring this aspect. We used the hierarchical clustering technique [23] because other clustering techniques, such as k-means, are used for variables (mainly continuous variables) for which the Euclidean distance is comprehensible. Given the type of data, hierarchical clustering analysis using binary distance (or Jacquard distance) seemed appropriate [11].

The distances between each pair of countries were calculated through the cluster analysis, such that groups were formed and associations were observed according to similarities. The resulting information was represented by a specialized graph called a dendrogram, which is highly useful for detecting associations. The value for the vertical cut-off point of the dendrogram was set automatically using the rect.hclust function from the stats library in R.

All the calculations and graphs were generated using the free statistical software R and R Studio (RStudio Team (2020). RStudio: Integrated Development for R. RStudio, PBC, Boston, MA URL http://www.rstudio.com/.)

## Results

Table 2 shows the types and numbers of officially recognized dental specialties in every country analyzed. Orthodontics and oral surgery were the most frequently recognized dental specialties in the analyzed countries (100% and 93.1% of the 29 countries with dental specialties, respectively). The total number of recognized dental specialties was 32, which was quite different among countries. Brazil had the highest number of specialties (23 dental specialties, 12 of which are only officially recognized there), followed by the United Kingdom (13), Australia, New Zealand, and the USA (12 each). However, Austria and Spain lacked officially recognized dental specialties at the beginning of this study.

**Table 2:**
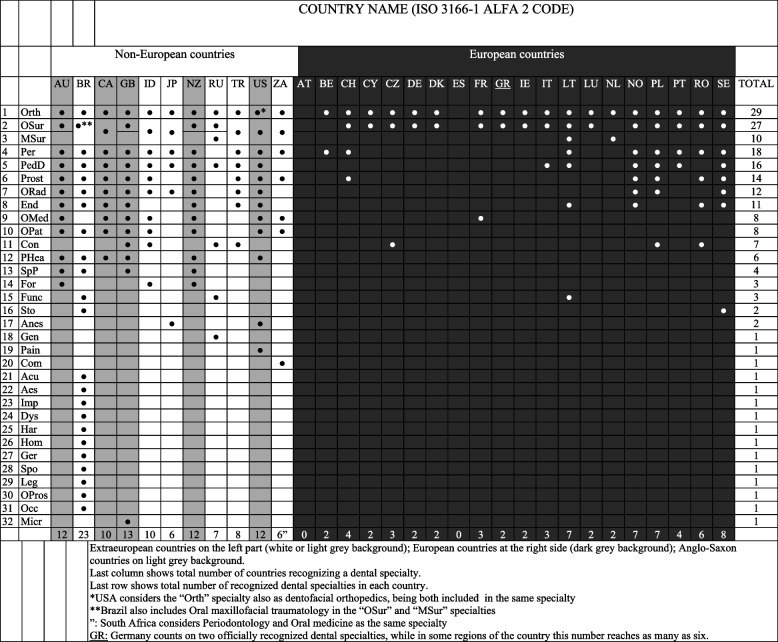
Recognized dental specialties in every analyzed country

Extraeuropean countries on the left part (white or light grey background); European countries at the right side (dark grey background); Anglo-Saxon countries on light grey background

Last column shows total number of countries recognizing a dental specialty

Last row shows total number of recognized dental specialties in each country

GR: Germany counts on two officially recognized dental specialties, while in some regions of the country this number reaches as many as six

*AU* Australia, *BR* Brazil, *CA* Canada, *GB* United Kingdom, *ID* Indonesia, *JP* Japan, *NZ* New Zealand, *RU* Russia, *TR* Turkey, *US* United States of America, *ZA* South Africa, *AT* Austria, *BE* Belgium, *CH* Switzerland, *CY* Cyprus, *CZ* Czech Republic, *DE* Germany, *DK* Denmark, *ES* Spain, *FR* France, *GR* Greece, *IE* Ireland, *IT* Italy, *LT* Lithuania, *LU* Luxembourg, *NL* Netherlands, *NO* Norway, *PL* Poland, *PT* Portugal, *RO* Romania, *SE* Sweden, *Orth* Orthodontics, *OSur* Oral surgery, *MSur* Maxillo-facial surgery, *Per* Periodontology, *PedD* Pediatric dentistry, *Prost* Prosthodontics, *ORad* Oral/dental maxillo-facial radiology, *End* Endodontics, *OMed* Oral medicine, *OPat* Oral pathology, *PHea* Public health, *Con* Conservative/restorative/therapeutic dentistry, *SpP* Special patients, *For* Forensic dentistry, *Func* Functional jaw orthopedics, *Sto* Stomatology/ stomatognathic physiology, *Anes* Anesthesiology, *Gen* General dentistry, *Pain* Pain in dentistry, *Com* Community dentistry, *Acu* Acupuncture, *Aes* Aesthetic dentistry, *Imp* Implantology, *Dys* Dysfunction of the temporomandibular joint and orofacial pain, *Har* Orofacial harmonization, *Hom* Homeopathy, *Ger* Geriatric dentistry, *Spo* Sports dentistry, *Leg* Legal dentistry, *OPros* Oral-maxillo-facial prostheses, *Occ* Occupational dentistry, *Micr* Oral microbiology

^a^USA considers the “Orth” specialty also as dentofacial orthopedics, being both included in the same specialty

^b^Brazil also includes Oral maxillo-facial traumatology in the “OSur” and “MSur” specialties

^”^South Africa considers Periodontology and Oral medicine as the same specialty

**Fig. 1:**
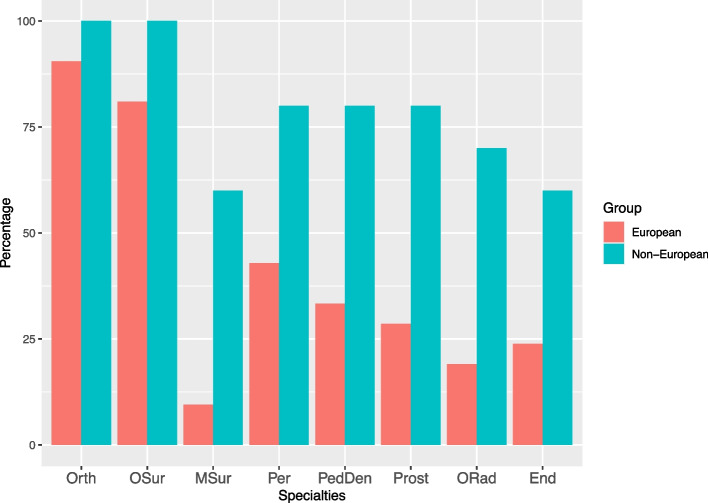
Percentage of the most frequent dental specialties in European and Non-European countries

Figure 1 provides comparative information regarding the percentages of the most frequent dental specialties in European and non-European countries, with a higher percentage in non-European countries. Differences of 50% or more were observed between European and non-European countries for maxillo-facial surgery, prosthodontics, and oral radiology.

**Fig. 2:**
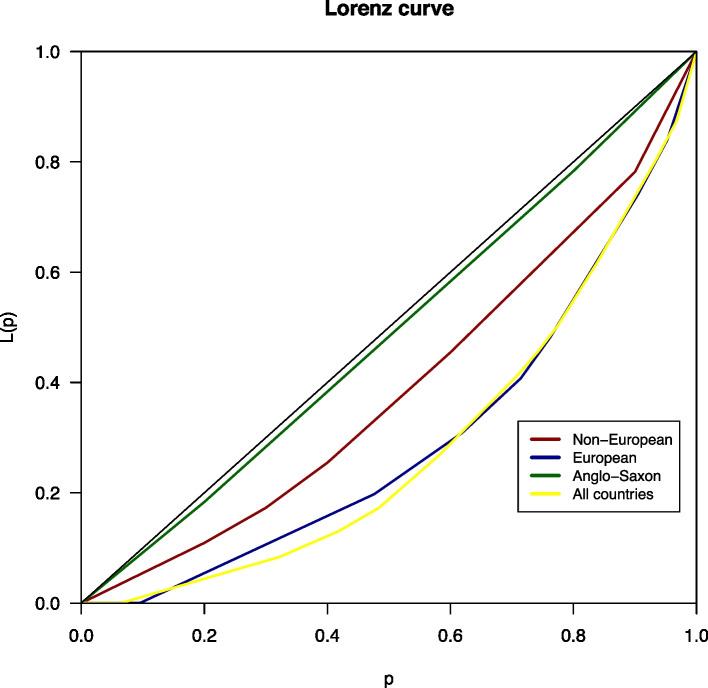
Lorenz curve of the Gini Index for each group of countries. In red non-European countries; in blue European countries; in green, Anglo-Saxon countries; in yellow, all the analyzed countries

The G index and Lorenz curve (Fig. 2) represent four groups of countries: European (in blue), non-European (in red), Anglo-Saxon (in green) (United Kingdom, USA, Canada, Australia, and New Zealand), and all countries (in yellow, G = 0.424). The inequality in dental specialties was almost double in European countries (G = 0.404) compared with that in non-European countries (G = 0.216). The green curve, of the five Anglo-Saxon countries, is the one that clearly covers the least area between the identity function and the curve itself, showing the lowest G index (G = 0.027). Anglo-Saxon countries had a G index value approximately 15-fold lower than that of European countries, thus showing the greatest similarity between them.

**Fig. 3:**
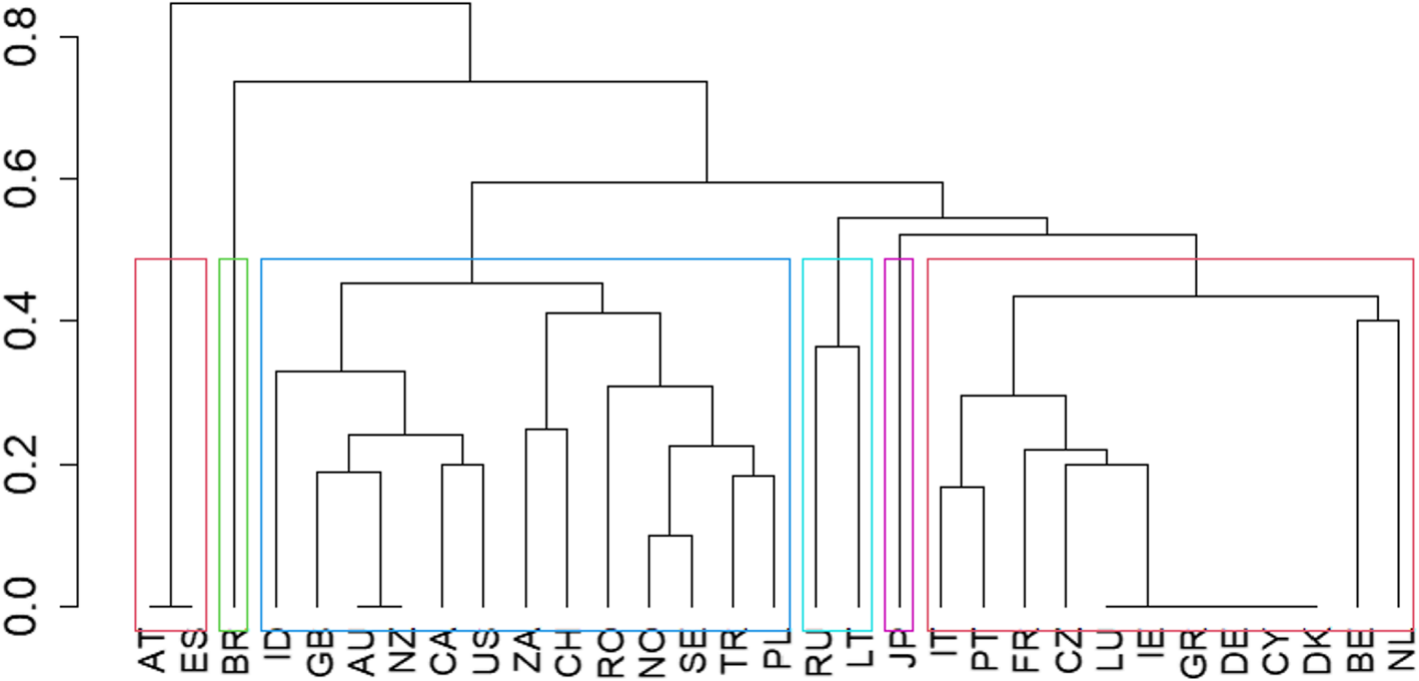
Dendrogram of the similarities between specialties of all countries. Country name according to ISO 3166–1 alpha-2 codes

The associated dendrogram of the cluster analysis (Fig. 3) is displayed as a forked-line diagram, where each fork groups countries at the lower level. The height of each fork indicates the maximum distance between the countries that formed it. To interpret our data, if we intersect the vertical axis, for example, at a distance 0.47, we will obtain six homogenous groups of countries in terms of the number and types of specialties.

The first group was formed by Austria and Spain, two countries without an official dental specialty. Brazil remained in an individual cluster and had the highest number of dental specialties. The third group, the largest, comprised European and non-European countries, having a subcluster with all the Anglo-Saxon countries and Indonesia and another subcluster of European and Asian countries with a certain geographical proximity plus South Africa. In the fourth group, Russia and Lithuania clustered together, both neighbor countries at a geographical level. Japan constituted the fifth group. The last group was composed entirely of European countries divided into two sub-clusters: the largest included six of ten countries with an identical pattern of dental specialties, because they only recognized orthodontics and oral surgery, and the smallest sub-cluster included only two European countries, Belgium and the Netherlands, which recognized orthodontics as a dental specialty, but not oral surgery.

## Discussion

The present study showed irregularities between different continents and countries worldwide in terms of dental specialties.

Orthodontics, the first dental specialty to be recognized, was the official dental specialty in every country. Orthodontics was the constant element for every country or pattern of dental specialty. Every country which recognized dental specialties did not fail to include orthodontics. Oral surgery showed a trend similar to that of orthodontics. Other dental specialties that are globally recognized and performed in clinical practice include periodontics (18), pediatric dentistry (16), prosthetics (14), oral/dental maxillo-facial radiology (12), and endodontics (11).

Orthodontics and functional jaw orthopedics showed clear differences in terms of organization of a specific dental specialty worldwide. In majority of countries, the orthodontics specialty merged both, including some parts of orthopedics, although it was not included as an officially recognized specialty. In the USA, both orthodontics and dentofacial orthopedics are included under the same specialty (Orthodontics, the “Orth” specialty). In contrast, the functional jaw orthopedic specialty is a separate officially recognized dental specialty in Brazil, Russia, and Lithuania, including different skills that cannot be classified under the orthodontics specialty.

Dental specialties were listed in the descending order; however, oral surgery and maxillo-facial surgery were grouped together because these two closely related specialties can be analyzed more easily, towing to the fact that some countries recognized both specialties, while others recognized just one of them. Therefore, the maxillo-facial surgery specialty could be classified under Medicine or Dentistry, depending on the country. Countries, such as Canada, Indonesia, South Africa, and the USA, considered oral and maxillo-facial surgery as the same dental specialty, and Brazil also considered oral and maxillo-facial trauma as the same specialty. In contrast, Australia, New Zealand, and a vast majority of European countries officially recognized oral surgery, but not maxillo-facial surgery, as a dental specialty. Finally, Russia officially recognized oral surgery and maxillo-facial surgery as two separate dental specialties. The clear exception is The Netherlands, the only country that recognized maxillo-facial surgery and not oral surgery. Notably, maxillo-facial surgery was commonly recognized in non-European countries (72.7%), while in Europe it was considered as a different dental specialty than oral surgery in Lithuania (5%).

Significant variations were also observed in South Africa, the only country which considered periodontics and oral medicine as the same dental specialty.

Additionally, there were significant geographical variations within a country. For example, Germany recognized orthodontics and oral surgery in a general way throughout the country, but also had a particular organization in smaller territories, such as federal states and cantons. These cantonal differences can result in new dentistry-recognized areas and specialties, such as public health, and the number of officially recognized dental specialties in some areas can reach as high as six.

We considered Ireland a European and not an Anglo-Saxon country, because this study was based on officially recognized dental specialties; therefore, Ireland was clearly affected by the European Union regulations. We considered the legal factors, rather than cultural or geographical factors, more important in contributing to Ireland’s grouping.

Notably, a large number of dental specialties exist worldwide. Among those, 12 exist in Brazil alone; however, the existence of the other 20 dental specialties is still surprisingly high. Although almost every country has its own healthcare system, legislation, and a variety of rules and regulations, some of the most frequently recognized dental specialties were common among the majority of the analyzed countries.

Regarding the patterns of dental specialties, the G index showed total variation for all the analyzed countries (G = 0.424). Therefore, the total global degree of inequality related to the number of dental specialties in the analyzed countries was 42.4%. The G index decreased to 0.404 and 0.216 when only European and non-European countries, respectively, were considered and reached a minimum value (G = 0.027) for Anglo-Saxon countries. Based on these values, the Anglo-Saxon countries had the greatest similarities with respect to dental specialties. However, the number of European countries was significantly higher than that of Anglo-Saxon countries, which may have influenced the final value of the index.

Hierarchical cluster analysis revealed six patterns in dental specialties. A critical analysis indicated that Brazil, because of its absolute singularity, similar to Japan, constituted isolated clusters, and Russia and Lithuania, because of their significant historical, geographical, and political relations, constituted a separate cluster.

The European countries were integrated into four clusters: the first included Lithuania and Russia; the second comprised countries that had not officially recognized dental specialties (Spain and Austria) yet; the third comprised 12 countries, of which 10 recognized between two (Luxembourg, Ireland, Greece, Germany, Cyprus, and Denmark) and three or four dental specialties (Italy, Portugal, France, and the Czech Republic), always including orthodontics and oral surgery; and two recognized two specialties but did not include oral surgery (Belgium and the Netherlands). Finally, the remaining European countries (Switzerland, Romania, Norway, Sweden, and Poland), which had a high number of dental specialties and were located in the eastern part of Europe, were clustered with Anglo-Saxon countries (United Kingdom, Australia, New Zealand, Canada, and the USA), Indonesia, South Africa, and Turkey. The presence of European countries belonging to the European Economic Area in four different clusters suggests that the common legislation (Directives 2005/36/CE and 2013/55/UE) is currently indefinite, and therefore allows for a wide range of variations.

In contrast, all Anglo-Saxon countries were included in the same cluster and are as close as possible to each other. This may be the reason for the lowest G index value for Anglo-Saxon countries in this study. Therefore, the Anglo-Saxon pattern of dental specialties is more consistent than the European one. Anglo-Saxon countries had a higher average number of dental specialties i.e., between 10 and 13, and shared a minimum of 10 specialties i.e., the 10 most frequent specialties found in this study.

Some inequalities might have originated because different countries recognize different dental specialties. Moreover, this study highlights the difficulties in accessing dental specialization, barriers to board certification [24], restrictions on working abroad to maintain the same dental specialization [25] and guaranties and a lack of control over the names of specialists among countries. These limitations should be resolved to maintain and equilibrate the quality of work and services for both dental professionals and patients. There should be an agreed list of competencies for each specialty to enhance harmonization and standardization [7]. A common pattern of dental specialties would make it easier to work abroad while maintaining the same specialization.

In specialization training, time is an important factor and one of the most common reasons for refusal of nostrification/recognition within the EU. The 2005/36/EC and 2013/55/EU directives set that full-time specialist dental courses shall be of a minimum of three years’ duration. So, the most common duration in Europe is just three years (Austria, Bulgaria, Croatia, Cyprus, Estonia, Finland, France, Greece, Hungary, Ireland, Italy, Latvia, Lithuania, Luxembourg, Malta, Poland, Portugal, Romania, Slovakia, Slovenia, Sweden), with some countries asking for another mandatory, previous year of general dentistry, as Germany or Switzerland, and Denmark asking for at least two years as a dentist,—and one of the two years must be with children—before you can apply for the orthodontic specialist program. In Belgium there is a sixth year, in addition to the five-year degree in dentistry and in order to access the profession as a general dentist and to further gain the required title of specialist in general dentistry. In Belgium the specialization programs in periodontics and orthodontics, last three years and four years respectively. The orthodontic specialization program tends to switch to a 4-year program, as currently happens in the Netherlands, Switzerland and Czech Rep.

The control and method of allocating places for specialization training is really different between countries, even between EU members, because European directives allow very different options so that specialized theoretical and practical dental training can be carried out “in a university center, in a treatment teaching and research center or, where appropriate, in a health establishment approved for that purpose by the competent authorities or bodies”. So, the involved institutions are multiple.

Consequently, in some countries this control is under the responsibility of the Universities. So, in Italy the number of seats is the responsibility of the department of the university concerned, which creates a "Scuola di specializzacione" as part of the postgraduate program within the university.

On the other hand, in many other countries this control is more central and related to Health Ministry. So, in Poland, places are allocated centrally, directly through the Ministry of Health, which accredits universities and private practices for this purpose and the entire training ends with a state exam. In a similar way, in France, the Regional Board of Health (ARS) reports the needs of each region, and it is the Ministry that decides upon the schools (currently 14) and seats (approximately 50–55 every year), having to finish, by also presenting a final exam.

Austria has a mixed model, where the number and allocation of dental specialists is surveyed by the Health Ministry too, but the Universities have the possibility to offer as many education seats as they decide and this is more or less an economic decision of each university, because the educational costs for specialization studies are not covered by the government. In Belgium, the Health Public Ministry set the schools (currently five in orthodontics) and number of dental specialists to be trained (approximately 13 every year in orthodontics), but the Faculties choose their candidates.

Only in some countries is the control more related to the regulatory body of the dentists. So, in Portugal, the Ordem dos Medicos Dentistas (OMD) recognizes the suitability of postgraduate degrees in orthodontics (180 ECTS, full-time training), five at the moment, three in public Faculties and two in private ones and it is the College of Orthodontics, made up exclusively of orthodontists trained in suitable departments, who proposes to the OMD how many orthodontists it can train, although not all of them take the national final exam.

The titles of master in orthodontics and orthodontic specialist, function alongside each other in Italy and some Scandinavian countries. What makes a master's degree different from the title of specialist is the lack of official recognition as a specialist in that country, a term that is usually restricted only to those with an official training. Certain traits such as a three-year full-time training are mandatory and easy to identify but that is not enough. The European directives additionally establish the need to be supervised by competent authorities or bodies. This is particularly problematic in countries like Spain, without inner regulation of dental specialties or Austria with a very recent regulation (approved in Feb 2023, operative since September 1st, 2023) which makes the automatic recognition of the title impossible in other countries and creates confusion to patients, who cannot discern who is a properly trained dentist as a specialist, because there is not an official list of specialists. Currently, most countries have public online information with up-to-date lists of specialists, but patients usually do not verify the official doctor´s credentials, due to a lack of knowledge.

Another clear trait of the quality of the training of a dentist as a specialist is the recognition by some other institutions that establish common programs, rules and parameters (number of students, student/teacher ratio, director qualifications, number of treated cases, facilities, end of specialization thesis, evaluation criteria, evaluating commission, etc.) to standardize specialty training in Europe. The most important one in orthodontics is NEBEOP (Network of Erasmus Based European Orthodontic Postgraduate Programs) and EFP (European Federation of Periodontology) in periodontics. Their standardization follows the European legislation and expand, in a very detailed way, the criteria to be fulfilled (i.e., 4800 h of training for orthodontists), but it is not mandatory for the practitioner to obey these criteria during their training specialty. Recently, the World Federation of Orthodontics (WFO) has updated the minimum orthodontic program requirements with guidelines to be used worldwide [26].

The European directives on the recognition of professional qualifications (2005/36/EC and 2013/55/EU) do not recognize any restriction of professional practice to general dentists. Only national regulations could set that type of limitation and it would have an exceptional character.

The availability to make a master's degree affects not only the quality of education and research as has been reported [8], but the stability of the dental market and the number of people in a particular specialty. The chosen institutions (Universities, Health Ministry, official colleges and councils, etc.) to control the dental specialization procedure could be a major determinant, and the important differences in the total numbers of general dentists and specialists and ratios to existing population between different countries, has to be highlighted. In our opinion the role of the scientific societies of specialists is currently underestimated and only in a few countries do they play a significant role. For example, in the Czech Republic two committees, one for accreditation of the specialization and the other one for education in dentistry, act as advisory bodies to the Ministry of Health, which governs the procedure. Both of them have nine members, two nominated by the Czech Dental Chamber, two by the Czech Orthodontic Society, two by the Universities and three by the Ministry of Health.

It is important to highlight that the published information related to dental specialties differs significantly between the 31 analyzed countries, depending on the way official documents or annual reports are published, the purpose of the main webpage related to dental institutions, the health system organization, or just the visual aspect of delivering public information. Additionally, there are significant variations in the content and names of several dental specialties in different countries, which make comparisons difficult.

## Conclusions

The status of officially recognized dental specialties in different continents and analyzed countries showed an asymmetric organization. The number, names, and skills of the officially recognized dental specialties in the 31 countries analyzed exhibited significant differences, showing inequalities in their organization. The Anglo-Saxon pattern of dental specialties showed greater equality than the European pattern. Orthodontics was the only constant element among the different patterns.

## Data Availability

All data generated or analysed during this study are included in this published article.
